# Toward whole-brain dopamine movies: a critical review of PET imaging of dopamine transmission in the striatum and cortex

**DOI:** 10.1007/s11682-017-9779-7

**Published:** 2017-10-25

**Authors:** Heather Liu, Yasmin Zakiniaeiz, Kelly P. Cosgrove, Evan D. Morris

**Affiliations:** 1 Department of Biomedical Engineering, Yale University, New Haven, CT USA; 2 Interdepartmental Neuroscience Program, Yale University School of Medicine, New Haven, CT USA; 3 Department of Psychiatry, Yale University, New Haven, CT USA; 4 Department of Neuroscience, Yale University School of Medicine, New Haven, CT USA; 5 Department of Radiology and Biomedical Imaging, Yale University, Yale PET Center, P.O. Box 208048, New Haven, CT 06510 USA

**Keywords:** Dopamine release, Neuroimaging, Kinetic modeling, Model limitations, Smoking, Stress

## Abstract

The mesocorticolimbic dopamine (DA) circuit, comprising the mesolimbic and mesocortical DA pathways, plays a crucial role in reward, cognitive control, and motivation. The positron emission tomography (PET) radiotracer, [C-11]raclopride, has been used widely to image DA receptors and DA changes in the mesolimbic pathway before and after pharmacological and behavioral challenges. In certain circumstances, properties of traditional kinetic models—used to analyze dynamic PET data—are not well-suited to describing the effects of stimulus-induced DA release. To combat model shortcomings, the authors have advanced a suite of models that characterizes PET data in the presence of time-varying DA release. We review select [C-11]raclopride studies of the striatum during cigarette smoking to illustrate the advantages of such models. DA receptors occur in lower density in the cortex than the striatum. This, as well as higher relative background signal, poses a serious challenge to quantitative PET of DA changes in the mesocortical system. Novel high affinity radioligands [F-18]fallypride and [C-11]FLB457 have been used to image mesocortical DA transmission. Models with time-varying terms may also hold the key to optimizing sensitivity to changes in mesocortical DA. As an illustration, we compare recent PET studies of the effect of stress on cortical DA release. Finally, we consider some challenges and strategies for further optimization of sensitivity of PET to stimulus-induced DA changes throughout the whole brain.

**Electronic supplementary material:**

The online version of this article (10.1007/s11682-017-9779-7) contains supplementary material, which is available to authorized users.

## The mesocorticolimbic dopamine circuit

Dopamine (DA) is an essential neurotransmitter for every-day brain functions including experiencing pleasure, regulating attention, and learning to control urges. There are two primary DA pathways in the brain that play fundamental roles in these functions – the mesolimbic and the mesocortical DA pathways. In the mesolimbic DA pathway, a rewarding stimulus, such as food or drug, stimulates DA neurons in the ventral tegmental area (VTA) leading to DA release in the ventral striatum (aka., nucleus accumbens). The mesocortical DA pathway also originates in the VTA, but produces DA release in the prefrontal cortex (PFC) (Swanson 1982). The mesolimbic pathway is critical for reinforcing motivated behavior (e.g., drug use), whereas the mesocortical pathway underlies emotional response and cognitive control, such as learning to *inhibit* drug use (Salamone and Correa 2002; Berridge 2007; Robinson and Berridge 1993; Schultz 2007; Dichter, Damiano, and Allen 2012).

Dysfunction in one or both corticostriatal DA circuits has been thought to underlie psychiatric diseases such as addiction, attention-deficit hyperactivity disorder, and schizophrenia (Dichter et al. 2012). However, the precise mechanisms of dysregulation in these disorders are currently unknown. Histochemical, lesion and microdialysis studies in rodent brains were first used to identify the DA pathways (Kebabian et al. 1972; Willuhn et al. 2010). Unfortunately, these experimental tools (1) are limited by low spatial and temporal resolution, (2) cannot be used to record DA transmission in multiple brain regions simultaneously and, (3) are unsuited for clinical populations. With advanced techniques for analyzing PET data, we may be able to measure both the spatial distribution and the time course of DA transmission in humans in vivo and relate the observed patterns to behavioral performance and/or clinical outcomes.

A story is emerging that *temporal patterns* of DA transmission in response to a stimulus encode important information that may be relevant for understanding drug addiction, and treatment. These patterns may differ by location. Volkow and Swanson linked temporal patterns of [C-11]-cocaine uptake—an indirect marker of elevated synaptic DA – to temporal patterns of subjective reports of “feeling high” and “drug craving” for cocaine (Volkow and Swanson 2003). Rapid elevation of DA has thus come to be associated with fast onset of drug high and drug craving. Based on microdialysis work in rats, it has been hypothesized that the partial nicotinic agonist, varenicline (“Chantix”) reduces the reinforcing properties of nicotine by eliminating sharp peaks in the DA response to nicotine (Rollema et al. 2007). Microdialysis and [C-11]FLB457 PET have been used together to examine DA transmission in the cortex of monkeys, leading to claims that DA transmission may be slow (“therapeutic”) in the cortex and fast (“addictive”) in the striatum (Jedema et al. 2014). These distinctions would be consistent with postulated functions of the mesolimbic and mesocortical systems, respectively.

Here, we review select PET papers reporting the effects of smoking or behavioral tasks on DA transmission. The papers help to illustrate the need for sophisticated modeling to properly detect and quantify the contribution of the DA signal to the PET data. These studies and further methodological refinements can lay the foundation for detailed characterization of both the mesolimbic and mesocortical DA loops. Better spatiotemporal characterization of DA transmission will provide a more complete picture of the workings of the dopaminergic circuitry. Our first illustration is based on work using [C-11]raclopride to image mesolimbic DA activity. The second illustration contrasts two reports of imaging behavior-induced DA transmission in mesocortical areas.

## [C-11]raclopride has been used extensively to image the striatum

[C-11]raclopride (RAC) is a DA D2/D3 antagonist that is used for imaging dopamine (DA) receptors in the striatum. RAC is well-characterized and widely-used to study changes in endogenous striatal DA levels. It has been shown to be well-suited, kinetically, to displacement by DA changes caused by amphetamine (Morris and Yoder 2007). In the 20 years since the groundbreaking study by Koepp and colleagues demonstrating DA release during a goal-oriented motor task (Koepp et al. 1998), there have been about 150 papers using RAC and PET to study drug- or behavior-induced DA changes (PubMed search terms: ‘dopamine release’, ‘raclopride’, ‘PET’, ‘human’). For a thorough review, with particular focus on DA response to behavioral and cognitive challenges, see Egerton et al. (2009).

The Egerton review also covers PET imaging of drug challenges, including nicotine. Our focus has been on smoking. Nicotine binds to nicotinic acetylcholine receptors (nAChRs) on DA neurons in the VTA to activate DA pathways which manage reward and reinforcement (Imperato and Mitchell 1986). Suppl. Table 1 lists studies from the last 15 years that investigated nicotine-induced DA release with RAC. Binding potential relative to non-displaceable tissue uptake (BP_ND_) (Innis et al. 2007), a measure of receptor availability, was used to estimate DA receptor level during each scan. The *change* in BP_ND_ (ΔBP_ND_) was used as a measure of DA release during smoking. In general, the studies found that BP_ND_ decreased due to the smoking stimulus. However, the magnitude of DA release was highly inconsistent, both across investigators and within the same group of investigators. The three studies (Table 1) performed by Brody et al. (Brody et al. 2004, 2006; Brody et al. 2010) can serve as an instructive case study for understanding the challenges of measuring stimulus-induced DA release with PET.

**Table 1:** Comparison of smoking studies using RAC performed by Brody et al.

Author/Journal	Cohort	Smoking stimulus	Data window	Findings
Brody et al. (2004), *Am J Psychiatry*	20 ND: 10 smoked, 10 abstained	1 cig. outside scanner 50 min post-injection	10 min	26–37% reduction in BP_ND_ in left VC, NACC, and left VP
Brody et al. (2006), *Arch Gen Psychiatry*	45 ND: 25 smoked, 10 abstained	1 cig. inside scanner 50 min post-injection	30 min	8.4 ± 13.8% reduction in BP_ND_ VC and NACC
Brody et al. (2010), *Psychiatry Res. Neuroimaging*	43 ND	1 cig. outside scanner 50 min post-injection	30 min	8.6 ± 1.6% reduction in BP_ND_ in VC and NACC

*BP*_*ND*_ binding potential, *ND* nicotine dependent, *cig* cigarette, *VC* ventral caudate, *NACC* nucleus accumbens

In each instance, investigators used an Equilibrium analysis (Watabe et al. 2000) to estimate BP_ND_ before and after the stimulus. The stimulus (smoking) was performed at 50 min during a break in scanning although the infusion of tracer continued throughout. In the first study, the investigators recorded a 26–37% drop in BP_ND_ (increase in DA) from before to after the smoking break in those who smoked. They employed apparently identical scanning and analysis procedures in the two follow-up studies. Curiously, they were unable to reproduce the same large change in BP_ND_ as measured in the first study; the latter studies found only 8–9% change in BP_ND_. What could be at the root of such an inconsistency?

## Time-invariant models fail to accurately describe smoking-induced DA release

**Fig. 1:**
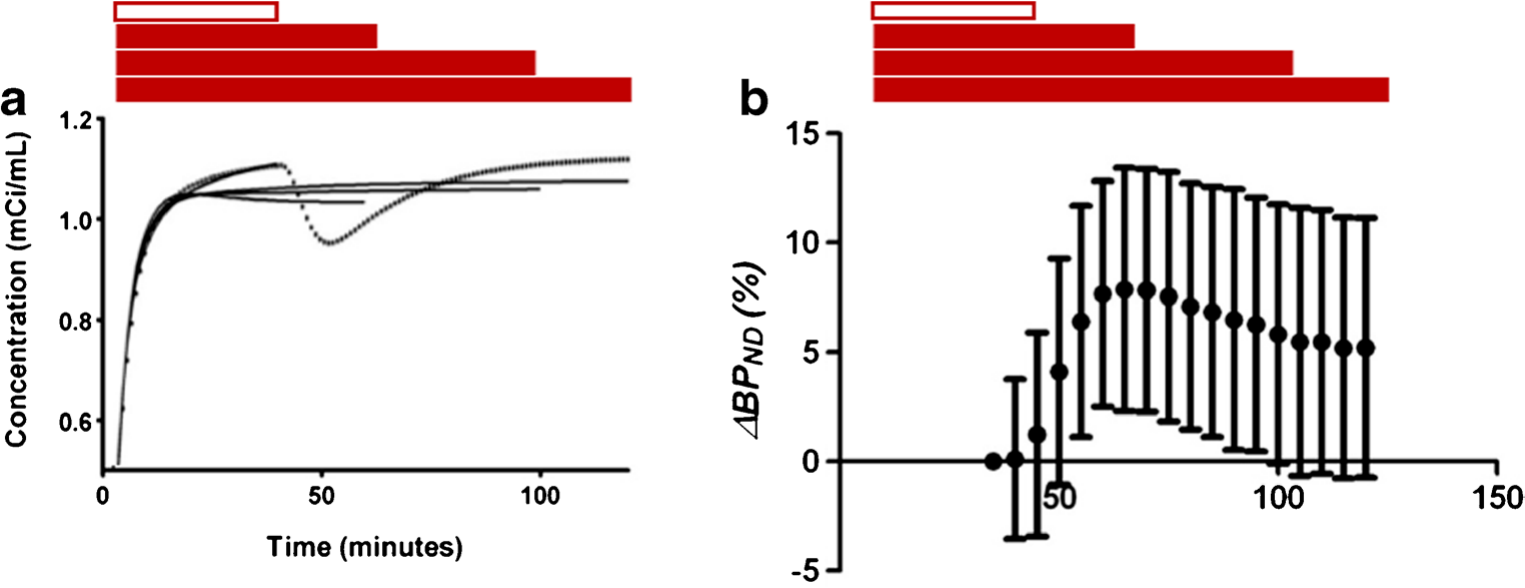
Simulated PET TACs including the effect of transient DA release. Hollow bars: data window for baseline fit, solid bars: data windows for fits including DA transient. (**a**) effect of data window on fitted curves with SRTM (solid: fitted curves, dotted: simulated original TAC). Note solid curve that fits the data perfectly *prior* to the effect of the transient at 40 min. (**b**) effect of data window on estimated change in BP_ND_ with SRTM, based on 100 simulated noisy TACs. Modified from Sullivan et al. (2013), *Am J Nuc Med Mol Imaging*

We have previously shown that inconsistency in the results of the above-mentioned smoking studies could be attributed to limitations of conventional models, such as SRTM, Logan plot, or Equilibrium analysis (Sullivan et al. 2013; Yoder et al. 2004). The calculated endpoint, BP_ND_, stipulates a *constant* level of DA binding throughout the duration of the scan. A consequence of applying the conventional models, which rely on time-invariant parameters, is that BP_ND_ becomes sensitive to the amount of data used post-stimulus. To demonstrate, Sullivan et al. simulated time activity curves (TACs) that incorporated a brief deflection indicative of transient DA elevation post-stimulus. Figure 1 demonstrates that inclusion of data during the transient DA elevation lowers the fitted curves relative to the baseline trajectory, producing substantial ΔBP – at least for narrow data windows. As the data window is widened to include recovery after transient DA release, the fitted curves are less responsive to the deflection and the estimated ΔBP is attenuated. Since the two follow-up studies performed by Brody et al. (2006, 2010) used a wider data window than the first study (30 vs. 10 min), it follows that the estimated ΔBP of the latter studies would be smaller.

## The linear parametric neurotransmitter PET model

The conflicting results catalogued in Table 1 (and Suppl. Table 1) indicate that ΔBP_ND_ may be an inconsistent estimator of smoking-induced DA release. Traditional models that estimate BP_ND_ do not contain explicit functions to describe short-lived neurotransmitter responses. By default, they assume that DA is constant—whether at baseline, or at some other value. Consequently, these models are unable to reliably capture transient responses (Yoder et al. 2004). The deficiencies in traditional models demand more flexible methods to fully characterize DA transmission. To resolve this deficiency, we have developed the linear parametric neurotransmitter PET (lp-ntPET) model (Kim et al. 2014; Morris et al. 2008, 2005; Normandin et al. 2012; Wang et al. 2016; Normandin and Morris 2006). Lp-ntPET is an extension of MRTM (Ichise et al. 2003).

1 $${{C_{T}}={R_{1}}{C_{R}} (t) + {k_{2}} \int\limits_{0}^{t} {{C_{R}} (u) du - {k_{2a}}} \int\limits_{0}^{t} {{C_{T}} (u) du - \gamma \int\limits_{\mathbf{0}}^{\mathbf{t}} {\mathbf{C}_{\mathbf{T}} (\mathbf{u}) {\mathbf{h}_{\mathbf{i}}} (\mathbf{u}) \mathbf{du}}}}$$

**Fig. 2:**
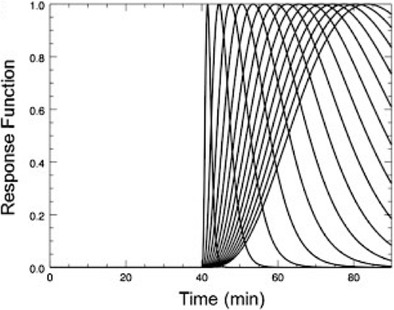
A partial set of gamma-variate shaped DA response functions as described by lp-ntPET. Modified from Kim et al. (2014), *Human Brain Mapping*

Lp-ntPET (Eq. 1) is the union of two models. The model is comprised of the left-hand term (conventional MRTM for tracer concentration, C_T_(t)), and the right-hand term in bold font (effect of time-varying DA, h_i_(t)). In practice, the optimal h_i_(t) is selected from a library of possible basis functions, which are typically configured as gamma variates but could take any form. Data from microdialysis studies support the use gamma variate functions for describing neurotransmitter release in response to stimuli Narendran et al. (2014; Tanda et al. 2015; Jedema et al. 2014). The gamma-variate functions used in lp-ntPET are each characterized by a distinct ‘sharpness (α)’, ‘response start time (t_D_)’, ‘peak response time (t_P_)’, and ‘peak height (γ)’. Figure 2 shows a representative (partial) set of bases that describe possible DA responses. Any of the conventional models would simply describe the DA response as a constant value for the entire scan.

DA release is deemed to be significant—and retained—only in those cases (voxels) for which inclusion of the final term in Eq. 1 containing the time-varying response function yields a better fit than the time-invariant model. Significance is determined by the F-test comparing the sum of squares from lp-ntPET to the sum of squares from MRTM (left hand term in Eq. 1 only). The F-statistic accounts for differences in degrees of freedom of the two models. That is, it penalizes the fit of lp-ntPET for additional model parameters.

Applied at the voxel level, the primary endpoint of lp-ntPET becomes a subject-specific spatiotemporal pattern of (significant) DA release over the course of the scan—which can be thought of as a ‘dopamine movie’. The action in a dopamine movie derived from RAC scans is confined to the striatum. Dynamic DA release can be detected with a temporal resolution on the order of minutes (Cosgrove et al. 2014; Normandin et al. 2012).

## Lp-ntPET is sensitive to subtle spatiotemporal fluctuations in DA during smoking

We have applied lp-ntPET to study the brain’s response to smoking Cosgrove et al. (2014). Eight male and eight female smokers were matched for smoking history and habits. The groups did not differ in radioactivity dose, injected mass of cold tracer, or craving prior to the scan. Overnight-abstinent subjects smoked inside the scanner, starting 35 minutes^1^ following the initiation of a bolus plus infusion of RAC. Correction for head motion was applied at the event-level during reconstruction (Carson et al. 2004). Lp-ntPET was applied in all voxels in the pre-commissural striatum (Kim et al. 2014).

**Fig. 3:**
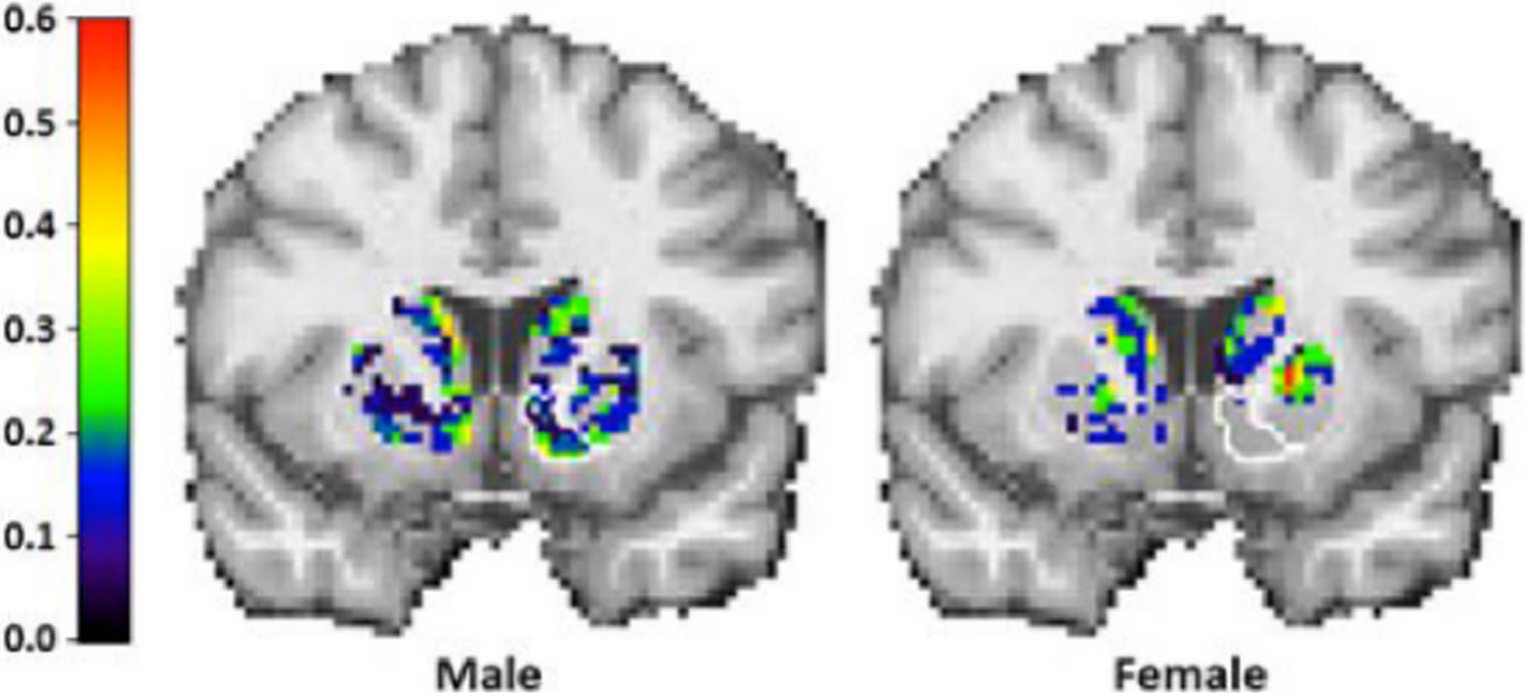
“Probability of Activation” maps for male (M) and female (F) smokers. Note difference between sexes in right ventral striatum (rVS). A permutation test performed using all 16 subjects showed mean difference in number of activated voxels in rVS between M and F was highly significant (*p* < 0.01). Right brain is on right. Modified from Cosgrove et al. (2014), *J Neurosci*

Significant sex differences were found in both the spatial extent and timing of activation during smoking (Fig. 3). Men activated consistently and robustly in the right ventral striatum while women did not. This finding is consistent with other research indicating that men smoke more for rewarding and reinforcing properties of nicotine compared to women (Perkins et al. 1996, 2002).

A consequence of lp-ntPET applied voxelwise is that the DA response can be visualized as a series of DA images over time. Online Resource 1 is a ‘movie’ of the DA response to smoking. Smoking began at 45 min for the subject; the response was estimated only in the pre-commissural striatum.


Online Resource 1“Dopamine movies” of DA activation for individual representative female (top) and male (bottom) smokers while smoking. The value at each voxel over time is the output of the lp-ntPET model. Four contiguous 2 mm coronal slices through the ventral striatum are shown for both subjects. Smoking started at 45 min. relative to injection of the tracer (0 min). The movie is comprised of 3 min frames from 25 min before smoking to end of scan. The color of the activation represents percentage increase in DA relative to baseline; percentage above baseline ranges from 0–150% (cool to hot colors). Note presence of activation in right ventral striatum in male, but not in female. The ventral activation in the male peaks from 65–70 min. The activation in the female is only in the dorsal striatal areas. Right side of brain is on the right. (MP4 263 KB)


The emergence of a significant finding of sex differences from a small cohort suggests that lp-ntPET is more sensitive to short-lived fluctuations in DA than traditional models. Smoking studies prior to Cosgrove et al. 2014 (Suppl. Table 1) could not provide any temporal information about the DA response. It appears that lpntPET can extract more nuanced information about DA release than was possible previously.

## High affinity tracers have been used to image cortical regions

The binding affinity of RAC (K_D_ ~ 1–10 nM) (Farde et al. 1986; Hall et al. 1988) is adequate for imaging D2/D3 receptors in areas with high DA receptor density relative to nonspecific uptake. However, RAC’s low signal-to-background ratio and low DA receptor density, extrastriatally, leads to unreliable quantification and limits use of RAC to the striatum (Egerton et al. 2009). Thus, RAC imaging can provide only a partial picture of the full corticolimbic circuitry.

Development of high-affinity radioligands, [F-18]fallypride and [C-11]FLB457, has made it possible to image DA receptors in areas of low DA receptor density such as the cortex (Mukherjee et al. 1999; Olsson et al. 1999). Human imaging studies within the past decade using [F-18]fallypride and [C-11]FLB457 have begun to elucidate cortical function during various DA-releasing pharmacological and behavioral challenges. A non-exhaustive list of these studies is given in Suppl. Table 2.

Some studies (Suppl. Table 2) used the Montreal Imaging Stress Task (MIST) to assess changes in DA in response to a behavioral stressor. MIST (Dedovic et al. 2005) is a well-validated model of psychosocial stress that requires the participant to complete mental arithmetic problems that are manipulated to prevent subjects from achieving their expected performance. This perceived underachievement is supplemented with “evaluative threats” via negative feedback and monitoring by investigators. Evaluative threats have been shown to significantly raise cortisol levels in participants (Dedovic et al. 2005).

## Studies using [F-18]fallypride and MIST provide a direct comparison of kinetic models

Table 2 provides details of two particular studies from Suppl. Table 2: Lataster et al. (2011) and Nagano-Saito et al. (2013). These studies together constitute an interesting comparison of methods. Both used [F-18]fallypride to image extrastriatal DA changes in healthy control subjects while performing MIST. Because the studies used the same tracer, stimulus, and a similar number of subjects, it would be reasonable to expect comparable results. The two research teams, however, applied different *kinetic models* with different calculated endpoints to their data: Nagano-Saito used SRTM (Lammertsma and Hume 1996) Lataster used LSSRM (Alpert et al. 2003). SRTM implicitly assumes no time-dependent change in DA. The calculated endpoint, BP_ND_, represents an average level of DA binding over the scan. Poor fitting of the data can lead to unreliable estimates of BP (see Sullivan et al. 2013). On the other hand, LSSRM allows for non-constant DA level during the scan. LSSRM models DA release as an exponential decay that peaks instantaneously at the start of the stimulus. The calculated endpoint, γ, represents the leading coefficient of the exponential, i.e. the peak DA level.

**Table 2:** Comparison of Lataster (2011) and Nagano-Saito (2013) by cohort demographics, experimental design, and analysis method

	Nagano	Lataster
Tracer	[F-18]fallypride	[F-18]fallypride
N	11 HC: 11 M	12 HC: 8 M, 4 F
Age	21.5 ± 3.3	38.8 ± 15.8
Stimulus	MIST	MIST
Smoothing (FWHM)	8 mm	4 mm
Mask location	Frontal gray matter	Prefrontal regions (BA9; BA10; BA11; BA24; BA32; BA44; BA45; BA46; BA47)
Search volume	207 mL	231 mL
MC correction method	Gaussian random field (FWER), *p* = 0.05	Simes-Hochburg (FDR), *p* = 0.05
Model/endpoint	SRTM, BP_ND_	LSSRM, γ

**Fig. 4:**
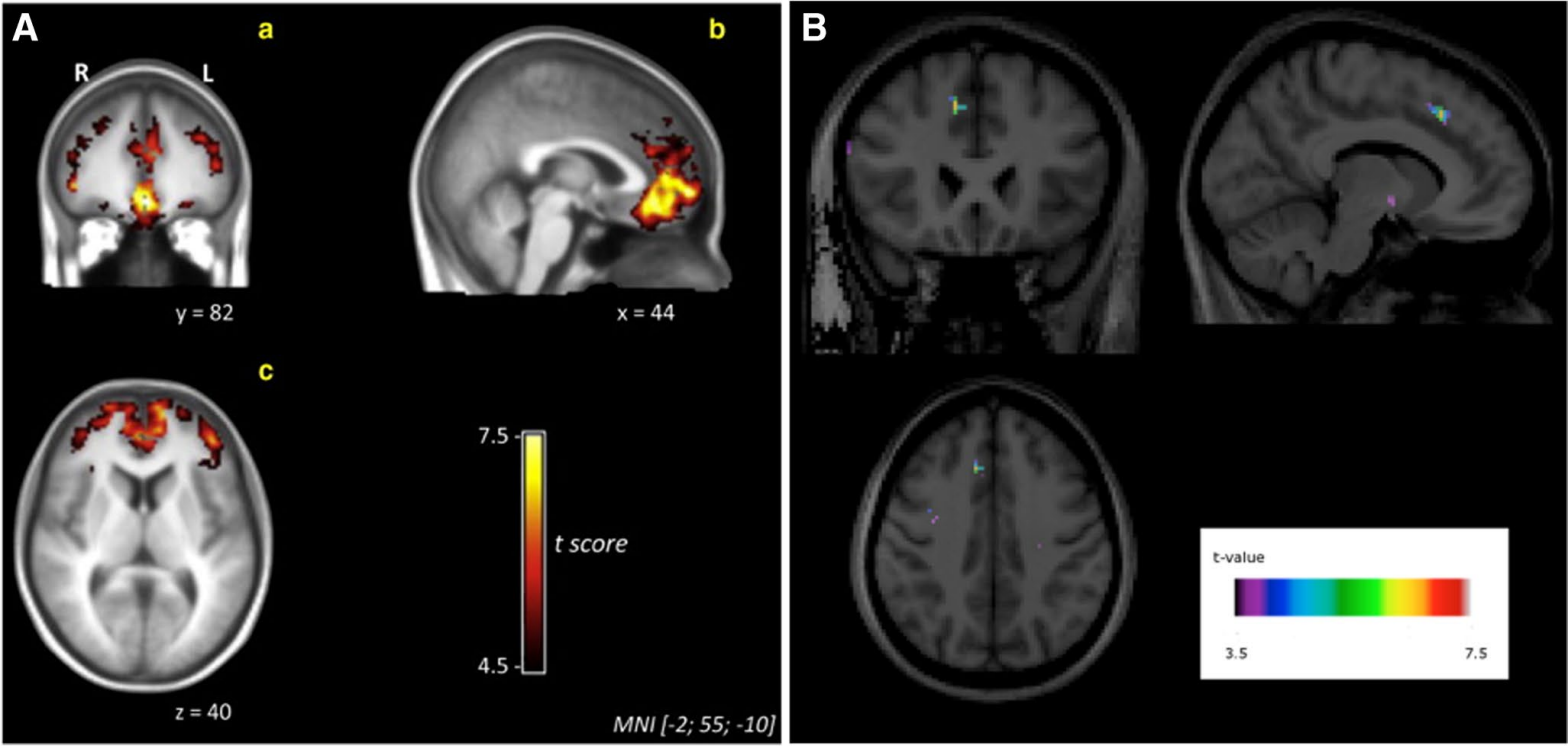
DA release during MIST. (**A**) t-score map based on γ as estimated with LSSRM from Lataster et al., (**B**) t-score map based on BP_ND_ as estimated with SRTM from Nagano-Saito et al. Used with publisher’s permission from Lataster et al. (2011), *Neuroimage* and Nagano-Saito et al. (2013), *Synapse*

Figure 4 is a side-by-side comparison of the t-maps published in the two studies. The t-scores were derived from parameters BP_ND_ and γ, estimated by SRTM and LSSRM, respectively. The t-scores represent effect sizes for DA release during the task, according to the two models. Despite similar experimental designs, the reader should note the considerable differences in the t-maps for the two studies. The effect sizes from the Lataster analysis are considerably greater than those of Nagano-Saito, as indicated both by the spatial extent of, and t-scores within the thresholded clusters. The differences in the strength of the findings lie in the differences in the capacities of the kinetic models to describe the data (Yoder et al. 2004). More robust detection of the effect by LSSRM over SRTM suggests that a time-varying model for DA release is the preferred descriptor of the data from a study using the MIST.

We acknowledge there are differences between the studies. Nagano et al. used a two-scan design in which control and stress scans were acquired on separate days. Lataster acquired control data and stress data within a single scan. There were slight differences in cohort demographics, data smoothing, and corrections for multiple comparisons. Despite these differences, it is most important to understand that the t-scores in Fig. 4 are uncorrected values. Thus, they can be compared directly to one another. Different methods of correcting for multiple comparisons may differentially affect the t-score *threshold* (determining significance) but the threshold would only affect the *spatial extent* of the clusters and not the *intensity values*. As can be seen in Fig. 4, the t-scores from LSSRM analysis are considerably higher than SRTM. In fact, there is minimal overlap between the ranges of t-scores from the two studies.

LSSRM appears to be more sensitive to transient DA than SRTM, but it may not be ideal for describing the widest range of possible DA responses. DA responses with unknown take-off or peak times will not be well-modeled. The limitations of LSSRM derive from its stipulations that (1) DA peaks instantaneously and (2) the shape of the DA curve is a decaying exponential. A model which allows the DA curve to take on a variety of forms—namely, allowing peak DA concentration to occur sometime after the start of the task—could be more widely applicable and potentially more robust. The lp-ntPET model (Eq. 1) fits this condition. In addition, lp-ntPET allows for varying response start times (t_D_), including negative t_D_. A negative t_D_ would indicate dopamine release starting before the start of the pharmacological stimulus. This might be the case if anticipation of reward were the dominant dopaminergic phenomenon. The application of lp-ntPET to cortical DA data is the next logical step to our goal of quantification of the spatiotemporal patterns of DA release in the entire corticolimbic circuit.

## Special considerations for creating whole-brain DA movies

The application of kinetic models that are not optimally suited to the PET data may result in high variability in calculated endpoints and diminished sensitivity for detection of stimulus-induced DA release. The deficiency in conventional models for describing data acquired by Brody et al. with consistency also likely explains the more robust findings from Lataster et al. (2011) compared to Nagano-Saito et al. The time-invariant model, SRTM, was unable to reliably capture the presumed time-varying DA component of the PET signal. On the other hand, the lp-ntPET model was able to characterize the spatiotemporal patterns of DA release during smoking with RAC PET Cosgrove, Wang et al. (2014). We are now preparing to extend the high sensitivity of lp-ntPET to create DA movies of the cortex—and ultimately the whole brain.

We anticipate challenges in the development of whole-brain DA movies. Simulations can be used in optimization processes as we did previously in the development of striatal DA movies (Wang et al. 2016). Microdialysis data of cortical DA (Narendran et al. 2014; Tanda et al. 2015; Jedema et al. 2014) can guide the creation of realistic simulations of extra-striatal DA responses. Simulations can also help to determine optimal binding kinetics for the tracer and optimal timing for the stimulus. Both are important for maximizing sensitivity (Wang et al. 2016). Full characterization of the corticostriatal DA response may require the use of RAC in the striatum and a high affinity DA tracer for the cortex. This, in turn, would require a means of synchronizing data from two scans sessions. Despite the challenges, we believe that ‘whole-brain DA movies’ will help us to visualize the entire corticolimbic circuitry and how its dysfunction might result in addiction.

## Electronic supplementary material

Below is the link to the electronic supplementary material.

Supplementary material 1 (DOCX 84.7 KB)

## Footnotes

1 Our protocol originally initiated smoking at 45 min. It was modified to 35 min, after we completed an optimization simulation study (Wang et al. 2016) that showed that sensitivity to DA is increased by an earlier stimulus. The optimal smoking time finds a balance between (1) acquiring sufficient baseline data before smoking to characterize tracer parameters, and (2) minimizing the effects of increased measurement noise in late-time data.
