# Supplementary material for: Toward whole-brain dopamine movies: a critical review of PET imaging of dopamine transmission in the striatum and cortex
Source: Brain Imaging Behav. 2017 Oct 25;13(2):314–22. doi: 10.1007/s11682-017-9779-7 (PMC5918462; doi:10.1007/s11682-017-9779-7)
Supplement: Supplementary file 2 — (DOCX 84.7 KB) [file 11682_2017_9779_MOESM1_ESM.docx]

# Title: Toward whole-brain dopamine movies: A critical review of PET imaging of dopamine transmission in the striatum and cortex

Heather Liu^1^, Yasmin Zakiniaeiz^2^, Kelly P. Cosgrove^2,3,4,5^, Evan D. Morris^1,2,3,5+^

^1^Department of Biomedical Engineering, Yale University, New Haven, CT

^2^Interdepartmental Neuroscience Program, Yale University School of Medicine, New Haven, CT

^3^Department of Psychiatry, Yale University, New Haven, CT

^4^Department of Neuroscience, Yale University School of Medicine, New Haven, CT

^5^Department of Radiology and Biomedical Imaging, Yale University, New Haven, CT

^+^ Corresponding Author

Address for correspondence: Evan Morris PhD, Yale PET Center, P.O. Box 208048, New Haven, CT 06510; Tel: 203 737 5752; evan.morris@yale.edu

| Author/Journal | Cohort | Smoking stimulus | Scan duration after stimulus | Model/endpoints | Analysis | Findings |
| --- | --- | --- | --- | --- | --- | --- |
| Brody et al. (2004), *Am J Psychiatry* | 20 ND: 10 smoked, 10 abstained | 1 cig. outside scanner 50 min post-injection | 10 min | Equilibrium, BP_ND_ | ROI-based | 26-37% reduction in BP_ND_ in left VC, NACC, and left VP |
| Brody et al. (2006), *Arch Gen Psychiatry* | 45 ND: 25 smoked, 10 abstained | 1 cig. inside scanner 50 min post-injection | 30 min | Equilibrium, BP_ND_ | Voxelwise | 8.4 ± 13.8% reduction in BP_ND_ VC and NACC |
| Barrett et al. (2004), *Synapse* | 10 ND: separate baseline/smoking scans for each subject | 1-6 cig. in 12 min intervals outside scanner, starting 15 min prior to injection | 60 min | SRTM, BP_ND_ | ROI-based | No significant change in BP_ND_ (from -57% to +70% in VS); ΔBP_ND_ correlated with hedonia |
| Scott et al. (2007), *Neuropsychopharmachology* | 6 ND, 6 HC | 2 denic. cig., 2 and 12 min post-injection + 2 cig. 40 and 50 min post-injection, both inside scanner | 40-50 min for denic. cig., 40 min for cig. | Logan Plot, BP_ND_ | Voxelwise | 10% BP_ND_ reduction from denicotinized to regular cigarettes in left VBG |
| Brody et al. (2010), *Psychiatry Res. Neuroimaging* | 43 ND | 1 cig. outside scanner 50 min post-injection | 30 min | Equilibrium, BP_ND_ | ROI-based | 8.6 ± 1.6% reduction in BP_ND_ in VC and NACC |
| Cosgrove et al. (2014), *J Neurosci* | 16 ND: 8 M, 8 F | 1-2 cig. inside scanner 35 or 45 min post-injection | 45 or 50 min | Lp-ntPET, γ, α, t_D_ | Voxelwise | Males activated (γ) consistently in right VS but females did not. Rise time (α) was consistently rapid for males but inconsistent for females. |

BP_ND_ = binding potential, ND = nicotine dependent, HC = healthy control, cig = cigarette, denic = denicotinized, VC = ventral caudate, NACC = nucleus accumbens, VP = ventral putamen, VBG = ventral basal ganglia, M = male, F = female

**Suppl. Table I** PET studies using [C-11]raclopride to image smoking-induced DA release. Table presents information about experimental design, data analysis, and findings from the studies

Barrett, S. P., Boileau, I., Okker, J., Pihl, R. O., & Dagher, A. (2004). The hedonic response to cigarette smoking is proportional to dopamine release in the human striatum as measured by positron emission tomography and [11C]raclopride. *Synapse,* 54(2), 65-71. doi:10.1002/syn.20066

Brody, A. L., E. D. London, R. E. Olmstead, Z. Allen-Martinez, S. Shulenberger, M. R. Costello, A. L. Abrams, D. Scheibal, J. Farahi, S. Shoptaw and M. A. Mandelkern (2010). "Smoking-induced change in intrasynaptic dopamine concentration: Effect of treatment for Tobacco Dependence." *Psychiatry Research: Neuroimaging*, **183**(3): 218-224.

Brody, A. L., M. A. Mandelkern, M. E. Jarvik, G. S. Lee, E. C. Smith, J. C. Huang, R. G. Bota, G. Bartzokis and E. D. London (2004). "Differences between smokers and nonsmokers in regional gray matter volumes and densities." *Biological Psychiatry* **55**(1): 77-84.

Brody, A. L., M. A. Mandelkern, R. E. Olmstead, D. Scheibal, E. Hahn, S. Shiraga, E. Zamora-Paja, J. Farahi, S. Saxena, E. D. London and J. T. McCracken (2006). "Gene variants of brain dopamine pathways and smoking-induced dopamine release in the ventral caudate/nucleus accumbens." *Arch Gen Psychiatry* **63**(7): 808-816.

Cosgrove, K. P., S. Wang, S. J. Kim, E. McGovern, N. Nabulsi, H. Gao, D. Labaree, H. D. Tagare, J. M. Sullivan and E. D. Morris (2014). "Sex differences in the brain's dopamine signature of cigarette smoking." *J Neurosci* **34**(50): 16851-16855.

Scott, D. J., Domino, E. F., Heitzeg, M. M., Koeppe, R. A., Ni, L., Guthrie, S., & Zubieta, J. K. (2007). Smoking modulation of mu-opioid and dopamine D2 receptor-mediated neurotransmission in humans. *Neuropsychopharmacology,* 32(2), 450-457. doi:10.1038/sj.npp.1301238

| Author/Journal | Tracer | N | Stimulus | Model/endpoints | Analysis | Findings |
| --- | --- | --- | --- | --- | --- | --- |
| Christian et al. (2006), *NeuroImage* | [F-18]fallypride | 8 HC | Spatial attention task | LSSRM, γ | Voxelwise | Activation (γ) in thalamus; γ correlated with task performance |
| Riccardi et al. (2006), *Neuropsuchopharmacology* | [F-18]fallypride | 14 HC | Oral amphetamine | SRTM, BP_ND_ | Voxelwise | Decreased BP_ND_ in striatum, substantia nigra, and cortex |
| Montgomery et al. (2007), *J Cereb Blood Flow Metab* | [C-11]FLB 457 | 12 HC | Oral methylphenidate | 2-TC, V_T_ | ROI-based | Decreased V_T_ in temporal and frontal cortical regions, and thalamus |
| Badgaiyan et al. (2009), *NeuroImage* | [F-18]fallypride | 8 HC | Emotional words | LSSRM, γ | Voxelwise | Activation in ligand displacement rate in left amygdala, left MTL, and left inferior frontal gyrus |
| Lataster et al. (2011), *NeuroImage* | [F-18]fallypride | 12 HC | Arithmetic stress task* | LSSRM, γ | Voxelwise | Activation (γ) throughout PFC; γ in vmPFC correlated with subjective stress |
| Vrieze et al. (2011), *Human Brain Mapping* | [F-18]fallypride | 10 HC | Monetary reward task | LSSRM, γ | Voxelwise | Activation (γ) in mOFC, vmPFC, dACC, γ correlated with reduced reward learning |
| Ray et al. (2012), *Neurobiology of Disease* | [C-11]FLB 457 | 14 PD: 7 PG+ 7 PG- | Gambling task | SRTM, BP_ND_ | Voxelwise | ΔBP _ND_ in midbrain was lower in PG+ compared to PG-; ΔBP _ND_ correlated with impulsivity |
| Nagano-Saito et al. (2013), *Synapse* | [F-18]fallypride | 11 HC | Arithmetic stress task* | SRTM, BP_ND_ | Voxelwise | Decreased BP_ND_ in small cluster in the dmPFC; ΔBP_ND_ correlated with HR |
| Lataster et al. (2014), *Schizophrenia Bulletin* | [F-18]fallypride | 10 HC, 14 FH PsyD | Arithmetic stress task* | LSSRM, γ | Voxelwise | First-degree relatives displayed attenuated activation (γ) in vmPFC |
| Milella et al. (2016), *J Psychiatry Neurosci* | [F-18]fallypride | 12 CD | Cocaine cues | SRTM, BP_ND_ | Voxelwise | Decreased BP_ND_ in mOFC and striatum; ΔBP _ND_ correlated with subjective craving |
| Jarcho et al. (2016), *NeuroImage* | [F-18]fallypride | 15 HC | Thermal pain | SRTM, BP_ND_ | Voxelwise | Decreased BP_ND_ in vmPFC during the placebo analgesic condition vs. water; ΔBP _ND_ correlated with subjective analgesic efficacy |

BP_ND_ = binding potential, HC = healthy controls, PD = Parkinson’s disease, PG = pathological gambler, FH = family history, PsyD = psychotic disorder, ND = nicotine-dependent, CD = cocaine-dependent

* Montreal Imaging Stress Task (MIST)

**Suppl. Table II** PET studies using [F-18]fallypride and [C-11]FLB 457 to image task- and drug-induced DA release. Table presents information about experimental design, data analysis, and findings from the studies

Badgaiyan, R. D., Fischman, A. J., & Alpert, N. M. (2009). Dopamine release during human emotional processing. *NeuroImage*, 47(4), 2041-2045. doi:http://dx.doi.org/10.1016/j.neuroimage.2009.06.008

Christian, B. T., Lehrer, D. S., Shi, B., Narayanan, T. K., Strohmeyer, P. S., Buchsbaum, M. S., & Mantil, J. C. (2006). Measuring dopamine neuromodulation in the thalamus: using [F-18]fallypride PET to study dopamine release during a spatial attention task. *NeuroImage*, 31(1), 139-152. doi:10.1016/j.neuroimage.2005.11.052

Jarcho, J. M., Feier, N. A., Labus, J. S., Naliboff, B., Smith, S. R., Hong, J. Y., . . . London, E. D. (2016). Placebo analgesia: Self-report measures and preliminary evidence of cortical dopamine release associated with placebo response. *Neuroimage Clin*, 10, 107-114. doi:10.1016/j.nicl.2015.11.009

Lataster, J., Collip, D., Ceccarini, J., Haas, D., Booij, L., van Os, J., . . . Myin-Germeys, I. (2011). Psychosocial stress is associated with in vivo dopamine release in human ventromedial prefrontal cortex: A positron emission tomography study using [18F]fallypride. *NeuroImage*, 58(4), 1081-1089. doi:http://dx.doi.org/10.1016/j.neuroimage.2011.07.030

Lataster, J., Collip, D., Ceccarini, J., Hernaus, D., Haas, D., Booij, L., . . . Myin-Germeys, I. (2014). Familial Liability to Psychosis Is Associated With Attenuated Dopamine Stress Signaling in Ventromedial Prefrontal Cortex. *Schizophrenia Bulletin*, 40(1), 66-77. doi:10.1093/schbul/sbs187

Milella, M. S., Fotros, A., Gravel, P., Casey, K. F., Larcher, K., Verhaeghe, J. A., . . . Leyton, M. (2016). Cocaine cue-induced dopamine release in the human prefrontal cortex. *J Psychiatry Neurosci*, 41(5), 322-330. doi:10.1503/jpn.150207

Montgomery, A. J., Asselin, M.-C., Farde, L., & Grasby, P. M. (2006). Measurement of Methylphenidate-Induced Change in Extrastriatal Dopamine Concentration using [11C]FLB 457 PET. *Journal of Cerebral Blood Flow & Metabolism*, 27(2), 369-377. doi:10.1038/sj.jcbfm.9600339

Ray, N. J., Miyasaki, J. M., Zurowski, M., Ko, J. H., Cho, S. S., Pellecchia, G., . . . Strafella, A. P. (2012). Extrastriatal dopaminergic abnormalities of DA homeostasis in Parkinson's patients with medication-induced pathological gambling: A [11C] FLB-457 and PET study. *Neurobiology of Disease*, 48(3), 519-525. doi:http://dx.doi.org/10.1016/j.nbd.2012.06.021

Riccardi, P., Li, R., Ansari, M. S., Zald, D., Park, S., Dawant, B., . . . Kessler, R. (2005). Amphetamine-Induced Displacement of [lsqb]18F[rsqb] Fallypride in Striatum and Extrastriatal Regions in Humans. *Neuropsychopharmacology*, 31(5), 1016-1026.

Vrieze, E., Ceccarini, J., Pizzagalli, D. A., Bormans, G., Vandenbulcke, M., Demyttenaere, K., . . . Claes, S. (2013). Measuring extrastriatal dopamine release during a reward learning task. *Human Brain Mapping*, 34(3), 575-586. doi:10.1002/hbm.21456
